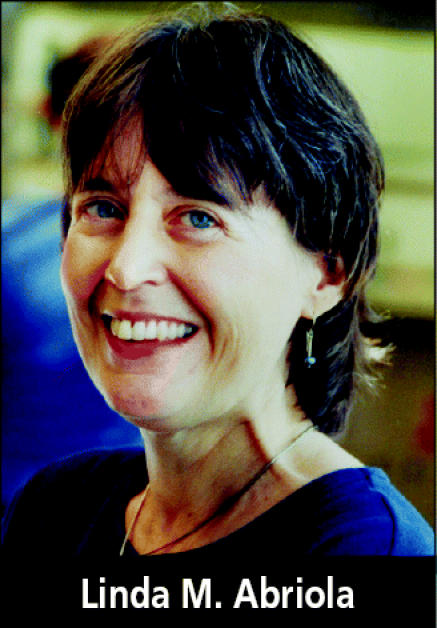# Guest Editorial: Contaminant Source Zones: Remediation or Perpetual Stewardship?

**DOI:** 10.1289/ehp.113-a438

**Published:** 2005-07

**Authors:** Linda M. Abriola

**Affiliations:** Department of Civil and Environmental Engineering, Tufts University, Medford, Massachusetts, E-mail: linda.abriola@tufts.edu

It has been some 20 years since I published my first paper on organic liquid contamination of the subsurface. That article was among the first to model the infiltration of organic solvents into aquifer systems. Before the mid-1980s, the importance of separate phase liquid pollutants was not appreciated, and most investigations into groundwater contamination had focused on solute (dissolved constituent) transport. Since that time, substantial resources have been dedicated to research on the behavior of what have become known as nonaqueous phase liquids (NAPLs). Within 5–10 years of those first articles, practitioners started to identify particular classes of NAPLs on the basis of their environmental persistence and ease of subsurface detection. The two most common classes are *a*) NAPLs composed of fuel hydrocarbons that are lighter (LNAPLs) than water and, thus, more easily detected, because they tend to remain within the unsaturated zone or capillary fringe areas of an aquifer; and *b*) organic solvent or dense NAPLs (DNAPLs) that tend to migrate deep into formations, becoming entrapped in irregular finger-like structures or pooled on low permeability strata. Laboratory evidence, coupled with a series of careful field case studies, soon revealed that fuel hydrocarbon plumes emanating from LNAPL contamination sites tended to stabilize with time, due to a series of microbial transformation processes (commonly termed “natural attenuation”). As a consequence of these investigations, monitored natural attenuation has become an accepted environmental management strategy for plumes at many LNAPL sites [e.g., U.S. Environmental Protection Agency (EPA) 1999; Wiedemeier et al. 1999].

For subsurface contaminant plumes that are attributable to organic solvent sources (of which estimates suggest there are as many as 25,000 in the United States alone), however, characterization and environmental remedy prescription have proven more elusive, and clean-up investments have often failed to deliver their promised outcome [National Research Council (NRC) 2005; Stroo et al. 2003; U.S. EPA 2003]. As I look back over the past two decades of research on DNAPLs, I am struck simultaneously by two realizations. First, we have certainly come a long way in improving our understanding of the migration, persistence, and recovery of DNAPLs in the subsurface environment. Second, we have failed to adequately incorporate this understanding into a long-term environmental management strategy. Below, I have attempted to summarize what I believe to be the most significant research findings and what I see as the most imposing challenges to implementing them. Although this editorial is devoted to the DNAPL problem, similar observations can also be made about any long-term contaminant source issue.

We now know that spatial variability in DNAPL mass distribution within a source region is almost inevitable, and, consequently, that mass detection is extremely difficult and uncertain [e.g., Interstate Technology & Regulatory Council (ITRC) 2003]. Migration pathways of DNAPLs will depend almost totally on the organic release characteristics (location, volume, composition, and rate), which are often unknown, and on small-scale subsurface textural variations that cannot be described deterministically (e.g., Kueper et al. 1993). Nevertheless, we have refined our conceptual and mathematical models of DNAPL migration to the point that we are confident in our predictions of migration pathways and equilibrium mass distributions for a fully characterized release scenario (e.g., Rathfelder et al. 2001e.g., Rathfelder et al. 2003). Furthermore, given a specific DNAPL distribution, we are also confident in our ability to model DNAPL dissolution, the process that will control source longevity and plume concentrations (e.g., Miller et al. 1998).

Given our current level of understanding, it has become clear that characterization of the source zone and the degree of uncertainty associated with that characterization are of critical importance in site assessment. Several DNAPL source zone characterization tools have been developed and demonstrated in the last few years (ITRC 2003). Although each of these has its potential uses, it is my personal perspective that a thorough delineation of mass distribution with these tools will not be feasible in the foreseeable future. However, reduction of uncertainty will likely be possible through the application of novel multistage characterization tools and protocols that incorporate knowledge obtained from initial characterization efforts into follow-on measurements (NRC 2005).

Considerable effort has also been directed toward the development and demonstration of so-called innovative remedial technologies. Many of these technologies involve flushing of the formation with various chemical amendments to achieve mass recovery or in-place mass destruction (e.g., surfactant flushing, *in situ* chemical oxidation). Others are predicated on creating *in situ* phase changes to facilitate mass removal (e.g., air sparging, six-phase heating). Although each technology has its proponents and some have been more fully refined than others, no single technology will work effectively under all conditions, nor will any technology be capable of achieving complete DNAPL mass removal and/or reduction of contaminant concentration levels to meet drinking water standards (NRC 2005). Because innovative technologies typically come with a large price tag and little guarantee of achieving regulatory end points, it is not surprising that there is a reluctance to implement these in the field. These issues, coupled with mass characterization difficulties, have led many to consider the DNAPL problem essentially intractable and to argue for containment as the best management strategy (Cherry et al. 1997; Freeze 2000).

Indeed, on an economic basis, simple cost/benefit analyses for remedial alternatives at DNAPL sites will typically lead to the selection of containment as a presumptive remedy. Few, however, seem to look beyond the 30-year present value cost horizon in such analyses. They also often fail to appreciate that the factors that limit the potential success of source remediation (insufficient characterization) may also limit the success of any proposed containment strategy. On the basis of simple mass partitioning calculations, one can derive estimates of typical DNAPL source zone longevities that span centuries under natural gradient conditions, or even longer time periods if hydraulic isolation is attempted. Such long time frames present an enormous challenge to site managers. Can we confidently guarantee adequate and consistent stewardship of physical or hydraulic containment strategies? Will the likelihood of containment failure and the costs of continued monitoring be factored into the cost/benefit analysis? Furthermore, will monitoring strategies be sufficient for timely detection of containment failure? A summary of 5-year reviews of existing physical and institutional controls under the U.S. EPA-administered National Contingency Plan suggests the answer to these questions is “no.” Although some progress been made since a 1995 U.S. EPA assessment, 5-year reviews continue to be characterized as paperwork exercises that result in few executable recommendations (Nakamura and Church 2000).

Perhaps the most promising news on the horizon is that, since the late 1990s, researchers have been identifying and isolating organisms and microbial consortia that are capable of transforming chlorinated solvents under a variety of subsurface conditions (Bradley 2003). Unlike the fuel-hydrocarbon scenario, however, the natural rates of these processes have typically proven too slow to handle contaminant loadings, and solvent plumes have been documented to persist and continue to expand for decades without apparent biotic attenuation. Fortunately, recent research suggests that coupling of innovative remedial technologies (partial mass removal) with biostimulation may lead to more effective remediation (Christ et al. 2005). For example, in a recent pilot-scale source zone remedial demonstration, our research group observed the stimulation of indigenous microbial populations after active flushing with a nonionic surfactant solution (Abriola et al. 2005; Ramsburg et al. 2004). This microbial activity resulted in the continued decline of source zone contaminant concentrations 450 days after active treatment. Although promising, much research is still needed to refine the design and explore the potential efficacy of such coupled treatment approaches.

The above observations lead to the conclusion that—from the standpoint of risk reduction—containment and perpetual stewardship as a DNAPL site management strategy is not easily justifiable. Even at sites where, at present, physical and chemical complexities permit no other viable management alternative, we routinely have failed to adequately estimate the uncertainty associated with the containment and monitoring plan. Uncertainty is difficult to quantify and thus often neglected. However, until we can evaluate the level of uncertainty associated with the observations and conceptual models upon which we base our site management decisions, assessing the cost and benefit of any characterization or remedial activity will be nearly impossible. Only after the development and employment of tools capable of quantifying uncertainty will we be able to assure the public that the actions taken are truly reducing risk. In tandem with the development of uncertainty tools, we must continue to pursue research into promising long-term source zone management strategies that couple aggressive remediation (mass removal or destruction) technologies with source zone biotic attenuation. Such a two-pronged approach promises substantial returns in the next 5 years.

## Figures and Tables

**Figure:**